# Identification of Early Signs of Mental Health Disorders in Older Survivors of Cancer Using Patient-Generated Health Data: Observational Study

**DOI:** 10.2196/75050

**Published:** 2026-06-12

**Authors:** Georgios Petridis, Antonios Billis, Georgios Meditskos, Athina Tsanousa, Paraskevas Lagakis, Juan Carlos Naranjo, José Zenóglio de Oliveira, Vania Tavares, Panagiotis D Bamidis

**Affiliations:** 1 Laboratory of Medical Physics & Digital Innovation, School of Medicine, Faculty of Health Sciences, Aristotle University of Thessaloniki, Building D, Entrance 8, 3rd Floor, Aristotle University of Thessaloniki Campus, Thessaloniki, 54124, Greece, +30 231 099 9237; 2 School of Informatics, Faculty of Sciences, Aristotle University of Thessaloniki, Thessaloniki, Greece; 3 Centre for Research and Technology Hellas, Thessaloniki, Greece; 4 MySphera, Valencia, Spain; 5 Capgemini (Portugal), Lisbon, Portugal

**Keywords:** behavioral data analysis, survivors of cancer, digital biomarkers, mental health monitoring, nonintrusive monitoring, patient-generated health data

## Abstract

**Background:**

Older survivors of cancer face heightened risk of depression and anxiety related to cancer experiences, fear of recurrence, and aging-related difficulties. Conventional mental health monitoring approaches, such as clinical assessments and even electronic patient-reported outcomes, are limited by recall bias, patient burden, and infrequent data collection. Emerging patient-generated health data from wearables and smart home devices offer passive, low-burden, continuous monitoring, but their ability to capture mental health risks in older survivors of cancer remains unclear.

**Objective:**

This study aims to explore whether patient-generated health data collected in the wild, either passively or actively, can classify older survivors of cancer as having or not having signs of anxiety and depression based on Patient Health Questionnaire-4 (PHQ-4) scores and to assess the potential added value of passive monitoring modalities, such as smart plugs.

**Methods:**

This study recruited 41 older survivors of cancer (mean age 72.3, SD 6.81 years) from the LifeChamps project. Over a 12-week period, participants were monitored using an activity tracker to measure physical activity, sleep, and physiological metrics; a smart scale to capture weight and body composition; and a smart plug to track television use as a proxy for sedentary television viewing. Mental health status was self-reported via the PHQ-4 questionnaire in a mobile app. Machine learning models were trained to classify mental health risk based on features derived from each sensor modality, both independently and in combination.

**Results:**

Tree-based gradient boosting models showed good performance in classifying PHQ-4–defined mental health risk. The best-performing configuration, combining smart plug and smart scale features, achieved a mean *F*_1_-score of 0.77 (SD 0.15) and a mean area under the receiver operating characteristic curve (AUC) of 0.85 (SD 0.10) across 3 repeated train-test splits. Standalone smart plug models, based solely on passive television use patterns, achieved a mean *F*_1_-score of 0.66 (SD 0.04) and a mean AUC of 0.71 (SD 0.06), outperforming models that relied only on activity tracker data (mean *F*_1_ 0.59, SD 0.2). Multimodal combinations tended to improve average performance but did not consistently yield large gains over the strongest single-modality configurations, likely reflecting adherence-related data loss for wearables and scales. Crucially, passive monitoring of television use patterns emerged as a promising behavioral proxy measure of mental health states.

**Conclusions:**

This study pioneers the use of passively collected data (eg, smart plugs) for mental health monitoring in older survivors of cancer, demonstrating their potential. Smart plugs capture behavioral patterns without user burden, with reasonable standalone performance (mean AUC 0.71, SD 0.06), positioning them as a promising low-burden modality. Future work should validate findings in larger independent cohorts and in prospective clinical workflows. Such technologies could transform monitoring for vulnerable populations, enabling scalable, inclusive care while reducing health care burdens.

## Introduction

### Overview

Older survivors of cancer face a unique set of challenges, including increased susceptibility to mental health issues such as depression and anxiety, 2 of the most common mental health disorders [1]. The intersection of aging, cancer survival, and mental health presents a complex clinical manifestation. These individuals often struggle with psychological distress stemming from their cancer diagnosis, treatment experiences, and the fear of cancer recurrence, all of which can significantly impact their quality of life (QoL) [2-4]. Furthermore, the combination of cancer recovery with aging processes can exacerbate mental health issues, making early detection and continuous monitoring critical for this demographic [5]. Emerging research underscores the importance of monitoring mental health to improve QoL among this demographic group, emphasizing the need for innovative approaches that are both effective and nonintrusive [6], so as to not exert additional burden on this vulnerable population.

### Background and Current Approaches

To address these challenges, the efficacy of using technological advancements for mental health monitoring has been widely explored in recent years.

A 2021 study [7] explored the potential of consumer-grade wearables, specifically Fitbit devices, to assess depression severity through digital biomarkers such as step count, heart rate, and sleep patterns. Their study, which focused on a cross-sectional working population in Singapore, demonstrated that wearable metrics could be significantly associated with depressive symptoms, with a sensitivity of 73% and specificity of 79%. This work underscores the feasibility of using common activity trackers to screen for mental health issues in general adult populations and presents a scalable model for remote monitoring of behavioral health indicators.

In an older adult population of 352 individuals, Lee et al [8] used low-cost activity trackers from Fitbit to monitor circadian rhythms and sleep patterns, finding correlations between these metrics and symptoms of depression and anxiety. This study highlighted that specific circadian rhythm markers, such as intraday stability and variability, can serve as predictors for geriatric depression and anxiety. The study’s findings supported the utility of passive, low-cost monitoring solutions for older adults, a critical demographic for interventions targeting mental health, by showcasing 69.8% accuracy for depression classifiers when using data only from the activity trackers.

Similarly, a study by Pedrelli et al [9] combined wearable (Empatica wristbands) and smartphone data to predict depressive symptom severity among 31 adults diagnosed with major depressive disorder. This research used multimodal data, including electrodermal activity, heart rate, and physical activity, as well as social engagement metrics from smartphones. The combination demonstrated that passive monitoring could reliably predict symptom changes, achieving high correlations between features and symptoms as well as a moderate mean absolute error between 3.8 and 4.7, emphasizing the potential of machine learning models in complex mental health assessments.

Furthermore, a systematic review [10] highlighted various studies that apply wearable sensors for passive data collection to detect symptoms of anxiety and depression. One of the significant findings was the identification of key behavioral markers detectable by wearable devices, such as heart rate variability, sleep disturbances, and physical activity levels, which consistently correlate with mental health conditions. However, the review highlighted a scarcity of research focused on older populations, where mental health burdens may intersect with age-related barriers to technology use. Additionally, Kim et al [11] recruited 20 participants aged 69‐90 years for a 90-day period, monitoring daily activities using passive infrared motion sensors. Their neural network-based classifier achieved more than 94% area under the receiver operating characteristic curve (AUC), suggesting that passive monitoring sensors can be effectively used to detect depression levels.

### Problem Statement

Despite the promising technological advancements outlined earlier, current standard-of-care methods for monitoring mental health still rely heavily on self-reported questionnaires and clinical assessments. These approaches present several challenges; they often rely on patients’ ability to recall, assess, and document their experiences, which can be hindered by memory recall biases, especially among older adults [12]. Additionally, the burden of frequent clinical visits and the adherence required for consistent self-reporting can be cumbersome, leading to reduced compliance and potentially delayed identification of mental health issues [13]. Even electronic patient-reported outcomes do not fully alleviate these burdens, as they still depend on active patient participation and do require an adequate level of digital literacy [14].

The sporadic nature of these assessment methods may fail to capture timely variations in mental health status, resulting in missed opportunities for early intervention. While the integration of wearable devices and smart home technologies offers a promising avenue to overcome these limitations [10], the efficacy of these technologies in accurately identifying mental health problems within vulnerable populations, such as older survivors of cancer, remains underexplored [15]. Specifically, the use of purely passive devices, such as smart plugs that monitor energy consumption and household appliance use, has not yet been thoroughly studied and remains an emerging topic [16].

### Objectives

To bridge this gap, this study aims to explore the added value that patient-generated health data (PGHD) collected in the wild, especially the ones that are passively collected, may contribute in terms of older survivors’ health-related QoL monitoring. Rather than performing a full clinical validation, we treat this work as an exploratory readiness assessment of whether machine learning models built on PGHD can meaningfully classify Patient Health Questionnaire-4 (PHQ-4)–defined anxiety and depression symptoms at each questionnaire occasion based on the preceding 30 days of sensor data, and what additional signal is contributed by different sensor modalities. In addition, this work attempts to compare the different modalities and their contribution toward the most accurate identification of mental health risk.

Specifically, this study contributes to the existing body of knowledge by:

- Evaluating the performance of machine learning (ML) models using data from smart plugs, wearables, smart scales, and their combination, in classifying the mental health status of older survivors of cancer.
- Comparing the discriminative contribution and potential added value of these unobtrusive monitoring modalities—individually and in combination—with particular emphasis on fully passive smart plug data as a low-burden option for longitudinal monitoring.

By demonstrating the discriminative value of ambient home sensors compared and in addition to wearables, this work offers evidence-based insights for integrating unobtrusive, cost-effective monitoring techniques into standard postcancer treatment surveillance and care [17].

In summary, our aim was to evaluate whether PGHD from smart plugs, activity trackers, and smart scales—alone and in combination—can classify PHQ-4–defined anxiety and depression symptoms in older survivors of cancer at the time of each questionnaire occasion based on the preceding 30 days of sensor data and to quantify the incremental discriminative signal contributed by fully passive smart plug data.

## Methods

### Study Design and Setting

This work is an observational study embedded in the Greek pilot site of the LifeChamps project [18], a multidisciplinary initiative designed to enhance the QoL for older survivors of cancer. The pilot involved home-based remote monitoring over approximately 12 weeks per participant using wearable devices, smart-home sensors, and a mobile app. Recruitment for this study took place between October 2022 and December 2023. All study procedures were conducted in collaboration with oncology and urology clinics in public and private hospitals or clinics in Greece, where patients were approached by their treating clinicians.

### Participants

Participants were recruited from the LifeChamps Greek pilot. Treating medical professionals (oncologists and urologists) identified potentially eligible survivors of breast and prostate cancer from their regular caseloads and invited them to participate during clinic visits.

The recruitment flow was as follows:

- Contacted: A total of 102 patients were approached (breast cancer: n=71 and prostate cancer: n=31).
- Consented: Of these, 50 (49%) patients (breast cancer: n=30 and prostate cancer: n=20) agreed to participate, provided written informed consent, and had the monitoring equipment installed at home.
- Excluded or refused: The remaining 52 patients either refused participation (n=35) or were deemed ineligible or unsuitable (n=17).
- Completed: Among the 50 participants who consented, 45 (90%) completed the full 12-week monitoring protocol.

For this analysis, we included the 41 participants who both completed follow-up and had at least 1 eligible postbaseline PHQ-4 assessment with corresponding sensor (smart plug) data. These 41 older survivors of cancer (female participants with breast cancer: n=24 and male participants with prostate cancer: n=17) had all completed their primary treatment and were either on adjuvant therapy or under surveillance.

### Inclusion and Exclusion Criteria

Eligible participants were older survivors of breast or prostate cancer under the regular care of the participating clinicians and living in the community. The specific criteria are presented in Textbox 1.

Textbox 1. **Eligibility criteria.**

**Inclusion criteria**

- Demographics: Age ≥65 years
- Cancer diagnosis or survivorship status: Histologically confirmed breast or prostate cancer, living beyond initial cancer treatment (surgery, chemotherapy, and/or radiotherapy), under adjuvant therapy or surveillance (as applicable), absence of diagnosed secondary malignancy
- Functional and cognitive status: Deemed physically and psychologically fit to participate (multidisciplinary team judgment); able to read, write, and understand Greek; Mini‑Cog score ≥ 3
- Technological requirements: Own Android smartphone (version 10+), 24/7 home internet access (Wi‑Fi and/or 4G; study provided mobile data where needed)
- Ethics or administrative: Able and willing to provide written informed consent

**Exclusion criteria**

- Clinical status: Terminal stage of cancer, prognosis <18 months from recruitment, currently receiving chemotherapy, active and unstable metastatic disease (clinician judgment)
- Mental or cognitive conditions impacting participation: Current diagnosis of a major mental disorder or cognitive disorder affecting the ability to participate
- Safety or device compatibility: Presence of an internally fitted medical device (eg, pacemaker), known allergy to metal or plastic that would preclude device use
- Ethics or administrative: Unwillingness or inability to provide written informed consent

### Ethical Considerations

The study protocol was reviewed and approved by the Research Ethics and Deontology Committee of the Aristotle University of Thessaloniki (protocol 267203; approval date: October 13, 2022). All participants provided written informed consent before installation of any sensors. The consent process described the types of data collected (including physical activity, sleep, physiological metrics, weight and body composition, and household appliance power consumption) and the duration of monitoring and clarified that participation was voluntary and could be withdrawn at any time without consequences for clinical care. To protect participant privacy, all sensor data were pseudonymized at the source and stored using unique participant codes; the linkage key was stored separately on a secure local server accessible only to authorized clinical staff. No identifiable images were collected or used in this paper or Multimedia Appendix 1. Participation was voluntary, and no financial compensation was provided; however, participants were allowed to keep the activity tracker or smart scale upon study completion as a token of appreciation.

### Data Collection

#### Overview

**Figure 1.**
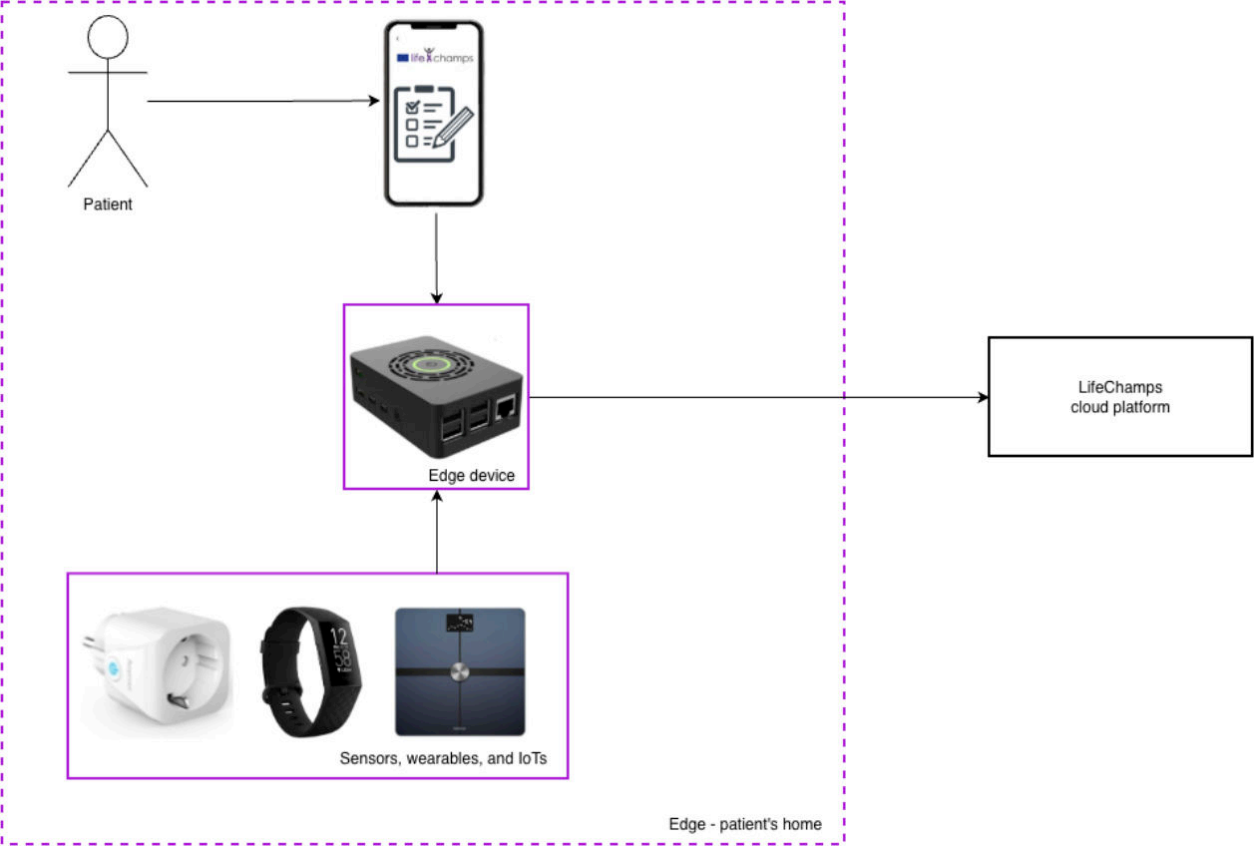
Overview of the LifeChamps home monitoring platform used in this observational study, showing the integration of Fitbit, Withings smart scale, and smart plug data with the central Edge device and data warehouse. IoT: Internet of Things.

The study spanned a 12-week period, during which participants’ homes were equipped with the LifeChamps Edge platform [19], a high-level overview of which can be seen in Figure 1. This platform included a suite of sensors and Internet of Things devices that tracked a breadth of activity and biometric data, constructing a comprehensive overview of each participant’s health and daily routine.

#### Activity Tracker

This wristband (namely the Fitbit Charge 4), worn consistently except during charging times, offered a stream of data covering physical activity (including duration, intensity, type, number of steps or floors, and calories burned), sleep metrics (including duration, efficiency, time in various sleep stages, time to fall asleep, sleep onset, wake time, and number of awakenings), and biometric parameters such as heart rate at different states (eg, during activities, resting heart rate, and sleep heart rate), and oxygen saturation levels. Data were synchronized with the vendor’s cloud service and then integrated into the LifeChamps platform.

#### Smart Scale

Participants engaged with the smart scale (namely the Withings Body+) on a weekly basis, which measured not only weight but also provided detailed body composition analytics, including muscle mass, fat mass, bone mass, and BMI. Unlike the continuous data stream from the Fitbit, the Withings scale provided discrete, yet vital, snapshots of the participants’ body composition over time.

#### Smart Plug

Integrated into the Edge platform and connected to the patient’s television (the main television used by them in case of multiple ones available), the smart plug (specifically the Teckin SP22) was used to monitor the television’s power status, recording on or off transitions every 5 minutes. This served as a proxy for one of the participants’ activities of daily living, offering insights into sedentary and leisure habits. Television watching is a sedentary behavior that has been associated with mental health status in prior work using validated psychometric tools [20].

In addition to sensor data, baseline demographic and clinical characteristics (eg, age, sex [male or female], marital and employment status, living situation, cancer type and stage, and Mini-Cog score [21]) were collected at study entry and later used as candidate features in the models. Participants also received a mobile app that served as the interface for self-report questionnaires, including the PHQ-4 mental health assessment described below.

### Mental Health Assessment

Participants in LifeChamps also received a mobile app, allowing them to self-report patient-reported outcome measures (PROMs) monthly. The PHQ-4 self-reported questionnaire [22] was used as the main self-report measure for mental health, assessing anxiety and depression with scores from 0 to 12, interpreted as normal mental health status (0‐2) or mild (3-5), moderate (6-8), and severe (9-12) symptoms of anxiety and depression. It includes 2 subscales for depression and anxiety, each total subscore ranging from 0 to 6, with subscores of 3 or above indicating symptom presence. PHQ-4 was selected as a compromise between more elaborate tools like Patient Health Questionnaire-9 and ultrabrief self-reports like Patient Health Questionnaire-2, based also on previous studies that included survivors of cancer and used the same questionnaire [23]. Although questionnaires like Patient Health Questionnaire-9 are not time-consuming on their own, in the larger context of the LifeChamps study where several PROMs were administered each month to a vulnerable group of people such as survivors of cancer, the decision was taken to opt in for brief versions of these PROMs whenever possible. On the other hand, the ultrabrief Patient Health Questionnaire-2 was not selected since it focuses only on depressive symptoms [24].

Participants were asked to report the PHQ-4 at 4 time points: at baseline and then monthly for 3 months. The first questionnaire was completed with researcher assistance, while subsequent ones were self-administered via app notifications at each specified interval. For this analysis, the baseline self-reports were not considered since they had no sensor data prior to their completion.

Each postbaseline PHQ-4 assessment, together with the corresponding 30 days of preceding sensor data, was treated as a single observation (questionnaire occasion) in the modeling analyses. Thus, the unit of analysis in this study is the PHQ-4 questionnaire occasion rather than the participant, and individual participants may contribute multiple observations over time. For the binary classification models, PHQ-4 total scores were subsequently dichotomized into “presence” versus “absence” of mental health signs, as described in the Data Preprocessing section.

### Data Preprocessing

We conducted a row-wise, participant-agnostic classification analysis to classify dichotomized PHQ-4 scores from demographics, questionnaire data, and sensor-derived features, treating each questionnaire plus its preceding 30 days of sensor data as a concurrent observation. As described earlier, each postbaseline PHQ-4 assessment, together with the corresponding 30 days of preceding sensor data, was treated as a single observation (questionnaire occasion). Because participants could contribute more than 1 questionnaire over time, multiple observations could originate from the same individual.

PHQ-4 scores were transformed to suit the needs of binary classification for the presence or absence of symptoms. This transformation was guided by clinical relevance and the nature of mental health progression in older survivors of cancer. Thus, all PHQ-4 PROMs with score 3 and above were categorized as “presence of mental health signs” to avoid any ambiguity, according to the suggested cutoff point for the Greek general population [25] and the one for patients with breast cancer [26], while the rest were assigned to class “no mental health signs.”

For each questionnaire occasion, we reconstructed the original feature blocks: (1) demographic and clinical characteristics (eg, age, sex, marital and employment status, living situation, Mini-Cog score, cancer type and stage, and treatment intent); (2) additional patient-reported outcomes (Brief Illness Perception Questionnaire, Medication Adherence Report Scale, Vulnerable Elders Survey-13, Edmonton Symptom Assessment System, and Linear Analog Self-Assessment); and (3) sensor-derived aggregates from the activity tracker (Fitbit), smart scale (Withings), and smart plug (television use). From the smart plug, we derived features such as daily total television on-time, daily off-time, and summary statistics (eg, mean and SD) across the 30-day window. We also experimented with features stratified by time of day (morning, afternoon, and night) and by weekday versus weekend; however, in the final models, we focused on simpler daily statistics, which yielded more stable performance in our experiments. To align with the monthly PHQ-4 assessments, we summarized daily and weekly sensor data into monthly aggregates. This process did not only involve simple aggregations, such as totals and averages, but also the computation of statistical measures that capture the variability and distributional characteristics of the data within each month. For each feature, we calculated the coefficient of variation to assess relative variability, the IQR around the median value, and the maximum and minimum values to encapsulate the range. Kurtosis was computed to understand the distribution, which can be indicative of behavioral patterns.

From the set of completed PHQ-4 questionnaires, we first excluded baseline assessments (which lacked prior sensor data and were used only for descriptive purposes) and any follow-up questionnaires without any smart plug data in the preceding 30-day window. To ensure that smart plug–derived features reflected meaningful television use patterns, we further excluded questionnaire occasions with fewer than 15 days of valid television data within the 30-day window or with 0 recorded television on-time during that period. As a valid day for the smart plug, we consider the days for which we have at least 10 hours of data. Fifteen days were selected as a threshold since they attribute 50% of the expected data within a month, a common threshold for considering missing values as manageable with imputation methods [27]. We selected smart plug data as a prerequisite, since the main aim of our analysis is to determine the efficiency of this completely passive monitoring modality as a standalone sensor and also compare it to the rest of the modalities.

Preventing data leakage, that is, avoiding any inadvertent use of information from the held-out test set during model training, was a priority, and as such, all algorithmic preprocessing steps were carefully applied to maintain the test set’s integrity. Automated feature selection techniques [28], such as importance thresholding, were used alongside clinical expertise and literature to inform the selection of features related to health-related QoL. To reduce the risk of overfitting, we removed columns that were direct identifiers (eg, patient ID), and we dropped ultrasparse features with more than 70% missingness within the filtered dataset before any imputation. For the remaining features, numerical variables were imputed using an iterative multivariate imputation procedure (IterativeImputer), while categorical variables (eg, sex, treatment intent, employment status, and living situation) were imputed using the most frequent category, and then, one-hot encoding was applied. All imputation steps were fitted on the training data only within each resampling split. To address class imbalance in the binary PHQ-4 outcome, we applied the synthetic minority oversampling technique (SMOTE) strictly within the cross-validation folds to prevent data leakage and overoptimistic performance estimates. The validation sets remained composed entirely of real, unaugmented patient data, ensuring that all reported metrics reflect performance on actual participants.

The resulting feature set was scaled using standardization, adjusting the data to have a mean of 0 (SD 1), so that features were on comparable scales for algorithms that are sensitive to feature scaling, without changing their relative information content.

The final dataset, a temporal alignment of Fitbit, Withings scale, and smart plug data with monthly PHQ-4 scores, was crucial for the effective identification of patterns by our ML algorithms. The different categories of features extracted from each sensor are presented in Table S1 in Multimedia Appendix 1.

### Modeling and Evaluation

We framed the task as a supervised binary classification problem, aiming to classify the presence or absence of mental health signs at each questionnaire occasion based on the preprocessed feature sets described earlier. The dichotomized PHQ-4 outcome (total score >2 vs ≤2) served as the target variable, and each PHQ-4 questionnaire plus its preceding 30 days of sensor data was treated as a separate sample. Thus, the models estimate how well PGHD can discriminate between questionnaire occasions with and without PHQ-4–defined signs within the same 30-day window, rather than predicting mental health status at a future time point.

Several model families commonly used for tabular clinical data were evaluated, including elastic net–regularized logistic regression, random forests, and HistGradientBoosting classifiers. All models were implemented in scikit-learn and combined with the same preprocessing pipeline (imputation, encoding, scaling, oversampling, and feature selection) applied in a consistent way across sensor configurations. Hyperparameters were tuned using small, predefined grids chosen to balance flexibility with the limited sample size.

To obtain more robust performance estimates and reduce dependence on a single train-test split, we used a repeated stratified train-test evaluation with an inner cross-validation loop for model selection. For each of 3 repetitions (3 different random seeds), we performed a stratified 80/20 split of the 52 samples into a training set (80%) and an independent test set (20%), preserving the class distribution. On the 80% training portion, we then carried out 10-fold stratified cross-validation to tune hyperparameters, with AUC used as the primary optimization criterion.

Within this inner cross-validation, the full preprocessing pipeline (including imputation, encoding, scaling, oversampling with SMOTE, and random forest–based feature selection) was fitted on the training folds only and applied to the corresponding validation folds, ensuring that no information from the held-out test set leaked into model training. After tuning, the selected model was refit on the full 80% training set and evaluated on the held-out 20% test set. This procedure was repeated independently for each seed and for each sensor configuration (single modalities and combinations), yielding 3 test-set performance estimates per configuration.

We assessed model performance using standard classification metrics: AUC, *F*_1_-score, precision, and recall. For each configuration, we report the mean performance on the 3 held-out test sets together with the corresponding SD across seeds (interrun variability). Hyperparameters were tuned using inner cross-validation, with AUC as the optimization criterion. Given the modest sample size (52 questionnaire occasions from 27 participants, with 21 “presence” and 31 “no mental health signs”), these analyses are intended as exploratory and hypothesis-generating, and model complexity as well as hyperparameter ranges were deliberately constrained to mitigate overfitting. No formal a priori sample size calculation was performed; the sample size was constrained by the LifeChamps pilot and was considered appropriate for exploratory, hypothesis-generating analyses. To mitigate overfitting with 52 questionnaire occasions (21 “presence” and 31 “absence” of signs), we restricted model complexity and used repeated resampling and nested cross-validation rather than more flexible model families.

### Missing Data

Missing data in this study arose from 2 main sources: participant nonadherence to the protocol and technical issues affecting data capture. For the PHQ-4 outcome, questionnaires that were not completed at a given time point were simply absent and were not imputed; only completed postbaseline PHQ-4 assessments with corresponding sensor data were included in the modeling dataset. As described earlier, we additionally excluded questionnaire occasions with no smart plug data or with fewer than 15 valid television days in the preceding 30-day window, because smart plug–derived features were central to our research question and the smart plug application programming interface did not allow backfilling of data after Edge-device downtime.

In the final analytic dataset (52 questionnaire occasions from 27 participants; 174 features after removing ultrasparse features >70% missingness), overall cell-level missingness was 32.5% (2943/9048). After applying the television coverage criteria, smart plug features were complete (0% missing), while missingness remained for demographics (17.3%), activity tracker features (33.4%), and smart scale features (53.6%).

For sensor-derived features, partial missingness was common, particularly for the activity tracker and smart scale, due to factors such as low digital literacy, forgetting to wear or charge the device, or not using the scale regularly. To ensure data quality, strict “validity criteria” were applied. A “valid day” for Fitbit data required >10 hours of wear time. Data from invalid days were not taken into account. In the preprocessing pipeline, ultrasparse features with more than 70% missingness were removed before modeling, and the remaining numeric and categorical features were imputed using the iterative and most-frequent strategies described earlier, fitted only on the training data within each resampling split. Because PHQ-4 served as the supervised learning target (label), we did not impute missing PHQ-4 outcomes; only completed postbaseline PHQ-4 assessments were included in the modeling dataset, as outcome imputation would require strong assumptions and could introduce bias if missingness was informative.

To explore the missingness mechanism in the final analytic dataset, we first note that Little’s global test for data missing completely at random (MCAR) can be numerically unstable when the number of variables greatly exceeds the number of observations, and its *P* values should be interpreted cautiously. We therefore supplemented this with pairwise MCAR 2-tailed *t* test diagnostics that compare the distribution of each observed variable between rows where another variable is missing versus observed (as implemented in commonly used missingness-exploration toolkits). In total, 2269 of 19,082 (11.9%) tests were significant at *P*<.05, exceeding the approximately 954 of 19,082 (~5%) expected under MCAR, which is consistent with structured (non-MCAR) missingness. This was expected because missingness occurred largely at the device or modality level—when a participant did not use the Fitbit or Withings scale during a given period, all features derived from that device were simultaneously absent.

Importantly, we did not find clear evidence that missingness was associated with the study outcome (PHQ-4 score>2). The prevalence of psychological distress did not differ significantly between questionnaire occasions with comparatively complete versus incomplete sensor data, nor did participant-level mean missingness show a significant association with outcome status (Spearman ρ=−0.24; *P*=.23). Sex (male or female) distribution was comparable between completeness groups (*P*=.87), whereas age differed (68.4 vs 71.9 years; *P*<.001), indicating that younger participants tended to have more complete device data. Together, these findings suggest that missingness depended on observable covariates and device-use behavior—patterns consistent with (though not proving) a missing at random mechanism.

## Results

### Participant Characteristics and Final Sample

A total of 41 participants met the analytic inclusion criteria (completion of follow-up and at least 1 eligible postbaseline PHQ-4 assessment with corresponding smart plug data). These older survivors of cancer included 24 female participants with breast cancer and 17 male participants with prostate cancer. Their baseline sociodemographic and clinical characteristics are summarized in Table 1.

**Table 1.** Baseline sociodemographic and clinical characteristics of the 41 older survivors of breast and prostate cancer enrolled in the LifeChamps Greek pilot (Greece, 2022‐2023).

Variable	Values
Sex assigned at birth, n (%)	
Male	17 (41.5)
Female	24 (58.5)
Age (years), mean (SD)	72.3 (6.81)
Employment status, n (%)	
Retired	20 (48.8)
Employed	2 (4.9)
Unemployed	1 (2.4)
Unknown	18 (43.9)
Cancer type, n (%)	
Curative	37 (90.2)
Metastatic	4 (9.8)
Living situation, n (%)	
Alone	4 (9.8)
Cohabitation	19 (46.3)
Unknown	18 (43.9)
PHQ-4^a^ baseline assessment, n (%)	
Healthy	33 (80.5)
Mild symptoms	8 (19.5)

a PHQ-4: Patient Health Questionnaire-4.

Following the process described in the previous section, out of 123 expected postbaseline monthly follow-up questionnaires (41 participants×3 monthly follow-ups), 93 were collected from 41 participants due to varying adherence to the protocol. The distribution of PHQ-4 classes based on the participants’ answers is shown in Table 2.

**Table 2.** Distribution of PHQ-4^a^ categories (normal, mild, moderate, and severe) across all completed postbaseline monthly follow-up questionnaires (n=93) among the 41 participants.

PHQ-4 class	Questionnaires in class, n (%)
0 (normal)	30 (32.3)
1 (mild)	49 (52.7)
2 (moderate)	10 (10.8)
3 (severe)	4 (4.3)

a PHQ-4: Patient Health Questionnaire-4.

**Figure 2.**
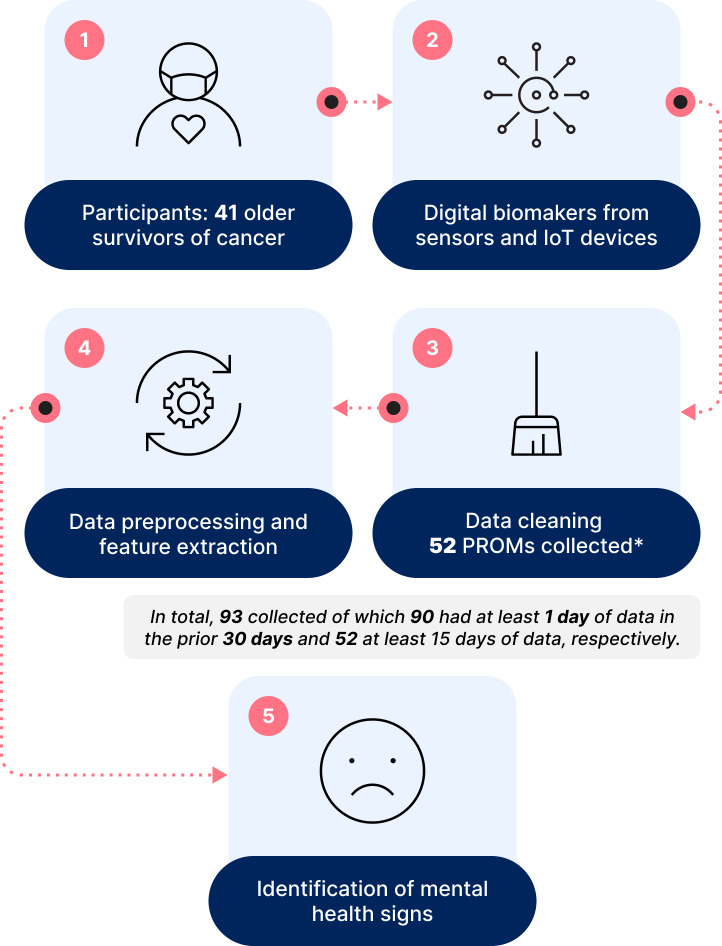
Analysis pipeline from raw sensor data and monthly PHQ-4 questionnaires to the final analytic dataset of 52 questionnaire occasions and training of machine learning models. IoT: Internet of Things; PHQ-4: Patient Health Questionnaire-4; PROM: patient-reported outcome measure.

After applying the preprocessing steps detailed previously, which are also presented in Figure 2, we derived a dataset comprising 52 questionnaire occasions contributed by 27 participants. Of them, 31 are categorized as “no mental health signs,” and 21 are identified as “presence of mental health signs.”

### Model Performance

Using the modeling approach described in the Methods section (3 repeated stratified 80/20 train-test splits with inner 10-fold cross-validation), we evaluated HistGradientBoosting models for each sensor configuration. Table 3 summarizes the mean test-set performance across the 3 held-out test sets, together with the corresponding SDs (interrun variability), for each configuration.

**Table 3.** Binary classification model results.

Sensors or features used and metric	Test set, mean (SD across 3 seeds)
Smart plug	
AUC^a^	0.71 (0.06)
*F*_1_	0.66 (0.04)
Precision	0.76 (0.08)
Recall	0.66 (0.03)
Activity tracker	
AUC	0.64 (0.14)
*F*_1_	0.59 (0.20)
Precision	0.60 (0.20)
Recall	0.59 (0.20)
Smart scale	
AUC	0.67 (0.24)
*F*_1_	0.67 (0.17)
Precision	0.67 (0.17)
Recall	0.67 (0.17)
Smart plug+activity tracker	
AUC	0.78 (0.14)
*F*_1_	0.74 (0.06)
Precision	0.74 (0.05)
Recall	0.74 (0.06)
Smart plug+smart scale	
AUC	0.85 (0.10)
*F*_1_	0.77 (0.15)
Precision	0.78 (0.17)
Recall	0.76 (0.14)
Activity tracker+smart scale	
AUC	0.73 (0.09)
*F*_1_	0.64 (0.04)
Precision	0.74 (0.10)
Recall	0.65 (0.03)
All sensors	
AUC	0.85 (0.15)
*F*_1_	0.76 (0.12)
Precision	0.78 (0.13)
Recall	0.74 (0.11)

a AUC: area under the receiver operating characteristic curve.

Across all models, the best-performing configuration was the combination of smart plug and smart scale features, which achieved a mean test AUC of approximately 0.85 (SD 0.10) and a mean *F*_1_-score of 0.77 (SD 0.15). The model using all 3 sensor modalities (“all sensors”) showed similarly high performance (AUC=0.85; *F*_1_=0.76), indicating that combining multiple data sources can provide reasonably accurate classification of PHQ-4–defined mental health signs in this small cohort.

Among single-modality models, smart plug–only and smart scale–only configurations performed comparably, with mean *F*_1_-scores of about 0.66 (SD 0.04) and 0.67 (SD 0.17) and mean AUC values of approximately 0.71 (SD 0.06) and 0.67 (SD 0.24), respectively. The activity tracker–only model showed somewhat lower performance (*F*_1_=0.59; AUC=0.64). Models combining smart plugs with the activity tracker (AUC=0.78; *F*_1_=0.74) or with the smart scale (AUC=0.85; *F*_1_=0.77) tended to outperform single-sensor models, suggesting that complementary information from different devices can improve classification in this dataset.

The comparative efficacy of the smart plug features is particularly intriguing when contrasted with the activity tracker–only model. The latter is widely regarded in the literature as capturing signals associated with mental health indicators [29]. Nevertheless, the standalone smart plug model’s performance suggests that behavioral data related to sedentary habits may provide a useful behavioral signal associated with PHQ-4–defined mental health risk in our cohort.

**Figure 3.**
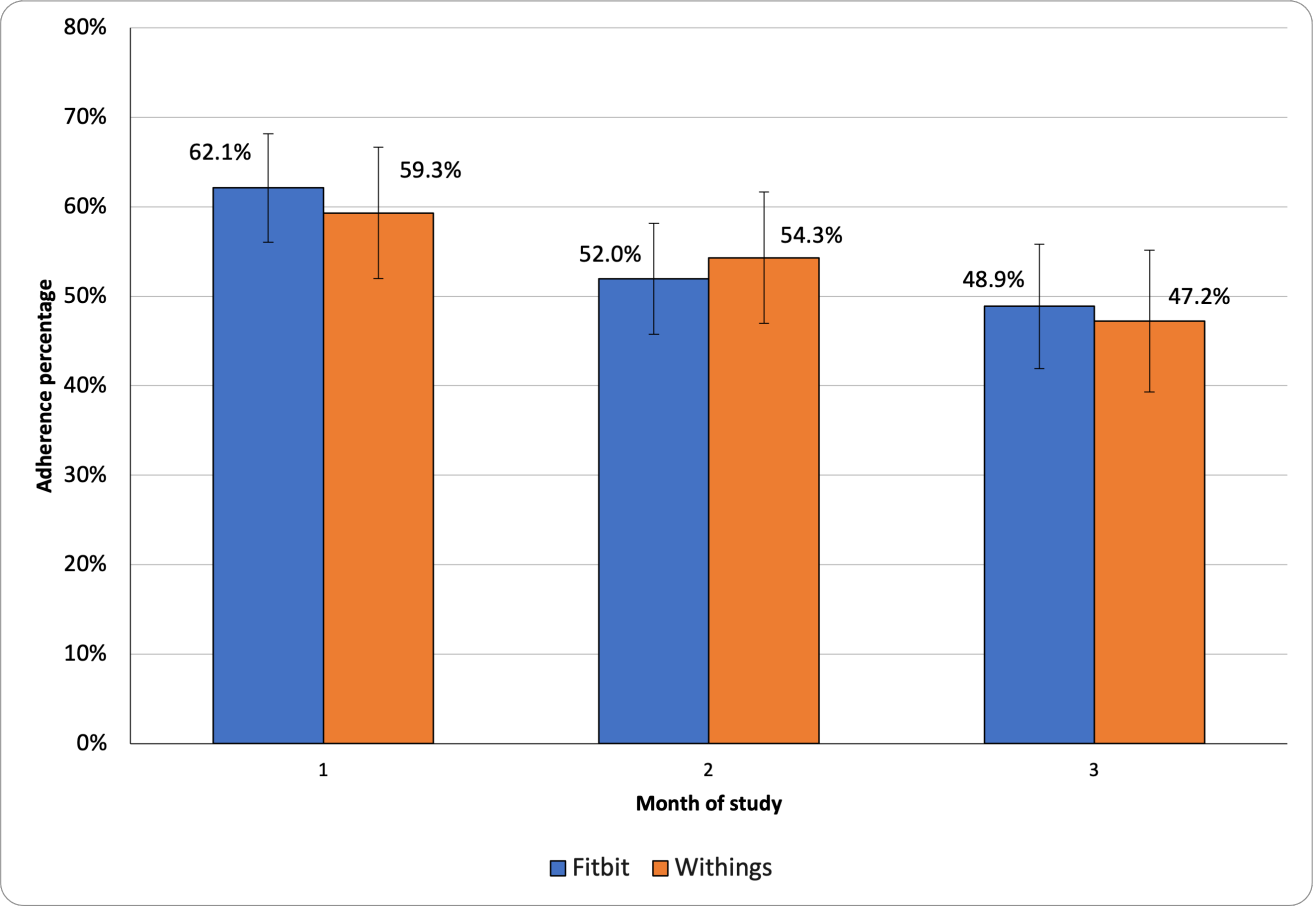
Mean adherence percentage of Fitbit (blue) and Withings (orange) through the 3 months of the study, in the original sample. Error bars represent the SE of the mean.

In the case of Fitbit’s and Withings’ data, the models exhibit moderate effectiveness with an AUC score of 0.64 and 0.67, respectively. These results indicate that, although these devices capture clinically relevant health metrics, their standalone discriminative value for classifying mental health risk appeared somewhat lower than that of models including smart plug features in this small sample. This difference may partly reflect declining adherence to wearing the activity tracker and using the scale regularly over the course of the study (Figure 3), which reduced the completeness and temporal density of the corresponding data streams.

Given the sample size and the variability observed across random splits, the reported performance estimates should be interpreted within the context of this experimental setup. They reflect how each modality behaves under the current data conditions and provide a consistent basis for comparing their relative performance.

## Discussion

### Principal Findings

This study explored whether PGHD collected in the wild can be used to classify PHQ-4–defined mental health signs in older survivors of cancer and to what extent completely passive smart plug data contribute to this task. Using a small sample of 52 questionnaire occasions from 27 participants, we found that models based on smart plug data, particularly when combined with smart scale measurements, achieved promising classification performance (test AUC around 0.85; *F*_1_-scores around 0.76‐0.77). Smart plug–only and smart scale–only models performed comparably to each other and better than activity tracker–only models, suggesting that television use patterns and weight or body composition metrics may contain informative behavioral signals associated with mental health risk in this cohort. These findings should be interpreted as preliminary and hypothesis-generating, but they support the idea that low-burden passive home sensing can contribute meaningfully to mental health monitoring in older survivors of cancer. Because both PGHD summaries and PHQ-4 scores refer to the same 30-day window, these analyses should be interpreted as cross-sectional discriminative associations between behavioral patterns and PHQ-4–defined mental health signs, not as prognostic models of future mental health trajectories.

Our results complement prior work on PGHD and digital mental health monitoring. Existing studies have shown that consumer wearables can capture behavioral and physiological markers associated with depression and anxiety, such as heart rate variability, sleep, and activity patterns [7-10]. Studies in older adults, including those using low-cost activity trackers or ambient motion sensors [8,11], have demonstrated associations between sensor-derived measures, circadian rhythms, and depressive symptoms, and some have reported high discrimination in small samples. However, most of these studies are either descriptive (focusing on correlations with symptom scores) or feasibility-oriented and rarely target older survivors of cancer specifically. By evaluating classification models that use smart plug–derived television use as a primary modality, alongside more conventional wearables and smart scales, our work extends this literature to a cancer survivorship context and highlights a novel, fully passive home-sensing signal.

The comparative performance of the different modalities provides some insight into their potential roles in real-world survivorship care. In our data, smart plug–only and smart scale–only models achieved moderate-to-good *F*_1_-scores (approximately 0.66‐0.67) and AUC values in the 0.67‐0.71 range, while the activity tracker–only model showed lower performance. When smart plug data were combined with either the scale or the activity tracker, model performance improved, especially for the smart plug+scale configuration. This pattern suggests that behavioral information about sedentary and leisure-time routines (television use) and anthropometric trends may complement each other for mental health risk classification, whereas activity tracker data in this sample may have been more affected by missingness and adherence constraints. We deliberately refrain from interpreting these differences as evidence that one modality is intrinsically superior; rather, they likely reflect a combination of signal content and data completeness in this specific cohort and setting.

From a clinical and implementation perspective, smart plugs and other passive smart-home devices have practical advantages for older survivors of cancer. Unlike wrist-worn activity trackers and connected scales, which require regular wearing, charging, and interaction, smart plugs operate entirely in the background once installed and configured. This low-burden data collection is particularly relevant for older adults living with multimorbidity, cancer-related fatigue, or low digital literacy, where even small self-management demands can reduce adherence. Our findings suggest that such devices can capture behavioral routines that are informative for mental health risk classification, and that combining them with minimally demanding periodic measurements (eg, weekly weight) may offer a feasible compromise between burden and information content. If validated in larger and more diverse samples, similar passive-sensing setups could be incorporated into survivorship follow-up to flag individuals who may benefit from more detailed assessment rather than to replace clinical evaluation.

Methodologically, we adopted a row-wise, participant-agnostic classification framework in which each PHQ-4 questionnaire and its preceding 30 days of sensor data constituted a separate observation. This choice allowed us to maximize the use of available data in a small sample, but it also means that multiple observations from the same individual are not strictly independent. In addition, we used SMOTE-based oversampling within the training folds to mitigate class imbalance and a repeated 80/20 train-test evaluation with inner cross-validation for model selection. Although these are commonly used techniques in the applied ML literature, there is active debate about the optimal handling of imbalance and the best validation schemes for small samples. Alternative approaches, such as participant-level cross-validation (eg, leave-one-participant-out and leave-one-group-out) and formal reclassification metrics (eg, net reclassification improvement and integrated discrimination improvement), were beyond the scope of this exploratory work but would be appropriate for future, larger-scale studies focused on model development and clinical validation.

### Limitations

We acknowledge limitations within our study. First, the sample size is modest (41 participants and 52 questionnaire occasions) and considering that participation required smartphone use and willingness to accept in-home monitoring, which may preferentially include more motivated and digitally literate individuals, leading to self-selection bias, performance estimates are therefore imprecise and sensitive to data partitioning. Despite using repeated stratified splits and reporting interrun variability, the reported metrics may overestimate or underestimate the true performance in other populations, and external validation in independent cohorts is needed. Second, the analytic dataset required sufficient smart plug coverage (≥15 valid television days in the preceding month), which means that we effectively conditioned the analysis on the availability of high-quality smart plug data. This design decision aligns with our primary objective—assessing the potential value of a fully passive modality—but may introduce selection bias and reduce direct comparability with models that could, in principle, be trained without smart plug data.

Third, missing data were frequent, especially for the activity tracker and smart scale, due to nonadherence (eg, forgetting to wear or charge the device) and technical issues. Although we used a principled preprocessing pipeline that removed ultrasparse features, applied imputation only within training folds, and avoided outcome imputation, residual missingness and imputation-related uncertainty remain. In addition, the smart plug application programming interface did not allow for backfilling data during Edge-device downtime, leading to irreversible gaps even when participants adhered to normal television use. In addition, PHQ-4 noncompletion at specific time points was treated as a simple absence of an outcome. We did not attempt to model “informative” missingness in questionnaire responses, and it is possible that individuals who felt particularly unwell or distressed were less likely to complete PROMs. Fourth, PHQ-4 was used as a brief self-report proxy for mental health symptoms rather than a clinician-administered diagnostic assessment, and it is therefore subject to recall and reporting biases. In addition, PHQ-4 has a 14-day recall window, whereas in this study, it was administered approximately monthly; this mismatch may blur transient symptom fluctuations and introduce measurement noise. We used a cutoff of 3 based on prior work in the Greek general population and breast cancer samples [25,26], but this threshold has not been validated specifically for older Greek survivors of cancer, and alternative cutoffs may yield different classification boundaries. Finally, television on-time is only a proxy for sedentary viewing; although a preliminary check using overlapping Fitbit step data suggested that participants were inactive during most television-on periods, we cannot exclude scenarios where other household members used the television while the participant was elsewhere in the home.

These limitations highlight the need for follow-up work along several directions. Future studies should aim to recruit larger and more diverse samples of older survivors of cancer, include independent external validation cohorts, and adopt participant-level validation schemes that more directly reflect prospective deployment. In addition to smart plugs and smart scales, other fully passive sensors (eg, ambient motion sensors and door or bed sensors) could be integrated to capture complementary aspects of daily routines, while maintaining low participant burden. On the outcome side, combining brief screeners such as PHQ-4 with more comprehensive, clinician-administered assessments in a subset of participants would enable more rigorous evaluation of classification performance and more robust calibration of clinically meaningful thresholds. Ultimately, the goal is not to replace clinical judgment but to evaluate whether unobtrusive, home-based PGHD streams can help identify older survivors of cancer whose mental health may be changing and who might benefit from more timely support.

### Conclusions

This study provides preliminary evidence that completely passive data collection, such as smart plug–based monitoring, may help identify mental health signs among older survivors of cancer, either as a standalone data source or in combination with other sensing modalities. The smart plug’s ability to capture granular, behavioral data without requiring patient interaction makes it a promising tool for passive health monitoring. The standalone efficacy of smart plug data highlights the importance of selecting specific, meaningful features over more comprehensive but potentially redundant data. Future research should aim to validate these findings in larger populations and explore the integration of additional nonintrusive sensors to further enhance the classification accuracy and robustness of mental health monitoring models in vulnerable population groups, such as older survivors of cancer.

By leveraging digital biomarkers and PGHD, this study paves the way for integrating smart home technology into regular health assessments. Such integration has the potential to transform care strategies for older survivors of cancer and reduce the burden on health care systems worldwide, making mental health monitoring more accessible and inclusive.

## Supplementary material

10.2196/75050 Multimedia Appendix 1 Overview of candidate features per modality.

## Abbreviations

AUC: area under the receiver operating characteristic curve
MCAR: missing completely at random
ML: machine learning
PHQ-4: Patient Health Questionnaire-4
PROM: patient-reported outcome measure
QoL: quality of life
SMOTE: synthetic minority oversampling technique
