# Supplementary material for: Identification of Early Signs of Mental Health Disorders in Older Survivors of Cancer Using Patient-Generated Health Data: Observational Study
Source: JMIR Cancer. 2026 Jun 12;12:e75050. doi: 10.2196/75050 (PMC13262779; doi:10.2196/75050)
Supplement: Multimedia Appendix 1 [file cancer-v12-e75050-s001.docx]

Table S1. Overview of candidate features per modality.

| Category | Features/Digital biomarkers (indicative) | Description |
| --- | --- | --- |
| Activity Metrics | Steps, Active Minutes, Calories Burned | Collected by Fitbit; measures physical activity levels and energy expenditure. |
| Sleep Metrics | Total Sleep Time, Sleep Efficiency, Time in Sleep Stages | Collected by Fitbit; details about sleep time and quality. |
| Biometric Data | Heart Rate, Resting Heart Rate, Oxygen Saturation | Collected by Fitbit; vital signs indicating physiological state. |
| Body Composition | Weight, BMI, Muscle Mass, Fat Mass, Bone Mass | Collected by Withings smart scale; detailed body composition measurements. |
| Behavioral Data | TV On/Off Status, Daily TV Usage Duration | Collected by Smart Plug; captures sedentary behavior via TV usage patterns. |
| Demographics | Age, Gender, Employment Status, Cancer type | Basic demographic information. |
